# Old World Vipers—A Review about Snake Venom Proteomics of Viperinae and Their Variations

**DOI:** 10.3390/toxins13060427

**Published:** 2021-06-17

**Authors:** Maik Damm, Benjamin-Florian Hempel, Roderich D. Süssmuth

**Affiliations:** 1 Department of Chemistry, Technische Universität Berlin, Straße des 17. Juni 135, 10623 Berlin, Germany; maik.damm@tu-berlin.de; 2 BIH Center for Regenerative Therapies, Berlin Institute of Health at Charité-Universitätsmedizin Berlin, (BCRT), 10117 Berlin, Germany; benjamin.hempel@charite.de

**Keywords:** venomics, proteomics, snakes, vipers, viperinae, toxins, venom, database

## Abstract

Fine-tuned by millions of years of evolution, snake venoms have frightened but also fascinated humanity and nowadays they constitute potential resources for drug development, therapeutics and antivenoms. The continuous progress of mass spectrometry techniques and latest advances in proteomics workflows enabled toxinologists to decipher venoms by modern omics technologies, so-called ‘venomics’. A tremendous upsurge reporting on snake venom proteomes could be observed. Within this review we focus on the highly venomous and widely distributed subfamily of Viperinae (Serpentes: Viperidae). A detailed public literature database search was performed (2003–2020) and we extensively reviewed all compositional venom studies of the so-called Old-World Vipers. In total, 54 studies resulted in 89 venom proteomes. The Viperinae venoms are dominated by four major, four secondary, six minor and several rare toxin families and peptides, respectively. The multitude of different venomics approaches complicates the comparison of venom composition datasets and therefore we differentiated between non-quantitative and three groups of quantitative workflows. The resulting direct comparisons within these groups show remarkable differences on the intra- and interspecies level across genera with a focus on regional differences. In summary, the present compilation is the first comprehensive up-to-date database on Viperinae venom proteomes and differentiating between analytical methods and workflows.

## 1. Introduction

Venoms are one of the major traits directly associated with snakes, however, only a small number of the over 3800 different species are highly venomous. Around 10% of all snakes belong to the viper family of Viperidae, which is grouped into three subfamilies with the clade of Azemiopinae and Crotalinae (‘pit vipers’) being sister to the Viperinae subfamily, the so called ‘true vipers’ or ‘pit-less vipers’ [1,2,3]. The evolutionary origin of Viperinae is still elusive, but dated to the middle Eocene and early Miocene 34–42 MA, with the oldest known viperine fossil (*Vipera antiqua*) found in central Europe [1,3,4,5,6]. From then onwards, they split into several lineages and conquered the ‘Old World’. Apart from a few exceptions, like Madagascar, Ireland, and several Mediterranean islands, these vipers can be found in South Africa, across Europe, the Middle East, up to Asia and even to the far eastern islands of Taiwan and Sakhalin. This vast distribution contributed to the name ‘Old World vipers’.

Each of the 101 true viper species (status: 31 December 2021; reptile-database.reptarium.cz) are venomous and in combination with their wide distribution range led to an increased number of encounters with humans. Especially in warm and densely populated regions of rural communities, interactions are not uncommon, and envenomation is a considerable burden to the public health. About 5.4 million snakebites are estimated to occur per year and while each second bite is a so-called ‘dry-bite’ and no venom is injected, the amount of venom that can be delivered in the other half varies due to several factors [7,8]. The size of the individual snake and thereby the highest possible volume of injectable venom, the kind of species, the time since the last meal, and other aspects are responsible for the outcome of these encounters [7,9,10,11]. Therefore, snakebite envenomation affect over 2.7 million people per year, which gives snakebites a great medical importance and concomitantly more global attention [12,13,14]. Among the most dangerous true vipers, in terms of highest mortality, are the African *Bitis arietans* (puff adder) and *Echis ocellatus* (West African saw-scaled viper), as well as two of India’s ‘Big Four’, namely, *Echis carinatus* (Asian saw-scaled viper) and *Daboia russelii* (Russell’s viper) [12,15]. Nevertheless, viperid bites are also a critical health issue in the Middle East and even Europe [16,17,18,19].

In recent decades, advances in bioanalytics facilitated deeper molecular insights into the composition of snake venoms, which constitute highly complex mixtures of proteins, peptides, and low molecular components [20]. Ultimately, the full venom composition is responsible for the different medical outcomes of snakebite envenomation [12]. It was shown that these multifaceted venoms differ not only between species but are also highly variable at an intraspecies level. Today, several factors are known to influence the venom composition of snakes [21,22]. One of the most important factors is the available prey and the accompanying diet breadth due to various habitats [23,24,25,26]. Furthermore, age as well as regional separation affect the venom, most likely linked to the available diversity of prey [27,28,29,30,31,32,33]. Sex, long-term captivity effects including stress [34,35,36,37], environmental conditions, like temperature, and the defense against primates are under discussion [38,39]. Venoms and their variations are of great scientific interest and can be seen as a model system for evolutionary biology, reaching from single genes to macroevolutionary contexts [40,41,42].

The investigation of venom diversity is strongly multidisciplinary, in which omics technologies, including genomics, transcriptomics, and proteomics, play an increasingly large role in the field of venom research [43,44]. Nowadays, the bottom-up (BU) and the top-down (TD) approach have become the gold standard in snake venom proteomics and the advantages and disadvantages of both have been extensively discussed [45,46,47,48,49,50]. The integration of high-resolution mass spectrometry (MS)-based workflows, mostly in combination with preceding decomplexation steps, plays a decisive role and has continuously developed over the past decades [51]. Today, de novo and database-dependent annotation methods allow the identification of toxin families, individual toxins, and various proteoforms requiring only minute quantities of venom [52]. In particular, the TD approach is on the rise and allows precise toxin identification directly from crude venoms and in this context the applicability of Fourier transform ion cyclotron resonance (FT-ICR) MS most likely will constitute a decisive step in the coming years [49,53]. While these methods only allow for a relative quantification of venom components, others like inductive coupled plasma (ICP) MS can be used for an absolute quantification, using the statistical abundance of cysteine sulfur in most venom proteins [54,55]. In addition, rather uncommon analytical tools have been used to investigate viper venoms, such as TD in-source decay (ISD) [56], venom on-a-chip [57,58], Fourier transform infrared spectroscopy (FTIR) [59], and the usage of a solid-phase combinatorial hexapeptide ligand library (CPLL) [60].

With constantly evolving technologies, the opportunities to investigate venoms faster and in more detail are surging. As a consequence, the number of new snake venom studies is growing rapidly every year. Some publications list several of those quantified venom proteomes, but Viperinae-related studies in particular constitute only a small part [61,62,63]. Although no up-to-date database summarizes all of these Viperinae studies in a comprehensive manner, there are a certain number of publications reviewing Viperinae venoms, that only focus on a few genera of high medical relevance or from exclusive areas [19,64,65,66,67,68]. However, there are many more studies on true vipers containing fully investigated venoms at the proteomic level. Therefore, we close this gap by providing a comprehensive compilation of recent venom compositions of Old World vipers and related compositional variations.

## 2. Viperinae Venoms: A Proteomic Database

For a detailed literature search of the Old World viper venom proteomes, we investigated contributions on all genera, species, and subspecies of the Viperinae subfamily up to the end of 2020. We included proteomic studies that analyzed the full venom, either by whole venom analysis or in combination with prior separation steps. In addition, the studies had to confirm the identity of the toxin or toxin family by MS or Edman degradation. For detailed workflows and selection criteria, we refer to the Methods section.

In total, we compiled 89 Viperinae venom proteomes from 54 studies, including 37 different species, belonging to 11 genera. The identified proteomes were analyzed and further classified to their kind of sequence annotation (bottom-up, top-down, Edman degradation), quantification method, and assigned toxin families (Table 1). The detailed database of all analyzed proteomes and values of the venom compositions are given in the Supplementary Materials Table S1. A composition therein corresponds to a single investigated venom or venom pool of a (sub)species within a study.

The number of reported venom proteomes per genus varies enormously and reveals that some genera are given more attention, while others are only analyzed once (Table 1). For example, well-investigated genera include the Oriental vipers *Daboia* with 24 venom compositions, followed by Palaearctic Vipers *Vipera* (18 compositions), and saw-scaled vipers *Echis* (16 compositions). Others like the bush vipers *Atheris,* with 18 species, only contain three venom proteomes in a single study [69]. Likewise, five out of 18 *Bitis* species venom compositions are known (Table 1). From the 13 Viperinae genera, three are monotypic: *Eristicophis* was analyzed only once, while the other two, *Montatheris* and *Proatheris*, are the only Old World viper genera not investigated until today [70]. Looking at the species level, the two viperid members of the ‘Big Four’ experienced the most venom proteome analyses: Russel’s viper *D. russelii* (12 compositions) and Indian saw-scaled viper *E. carinatus* (11 compositions). They are followed by the Eastern Russel’s Viper *D. siamensis* (9 compositions), a former subspecies of *D. russelii*. An additional 10 species were investigated twice or more for their venom composition and 23 venoms were described only once (Table 1).

**Figure 1:**
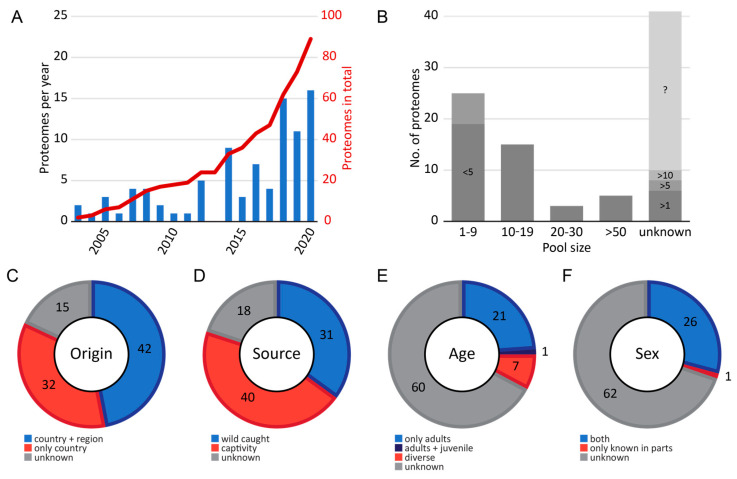
Meta data analysis for the 89 venom proteome studies of the Viperinae subfamily. (**A**) Overview of published true viper venom composition per year and the total number sum in red. (**B**) Variation of investigated pool sizes. Unknown ones in some cases were noted as a minimal or total unknown number. Given information in the reports of the investigated snakes about (**C**) the regional origin, (**D**) the source of the animal, like captured in the wild or kept in captivity, (**E**) age, and (**F**) sex. Detailed values and locations for each proteome are available in Supplementary Material Table S1.

The first published proteomic Viperinae venom proteome is from 2003 by Nawarak et al. and belongs to *D. siamensis* (published as *V. russelli siamensis* and *V. russelli formosensis*). The study reports on ten different vipers and elapids and employs a multitude of analytical techniques. Since then, each year (except for 2013) one or more new Viperinae venom proteomes was published with a steadily increasing trend (Figure 1A). In the past three years, one year after the reinstated status of snakebites as a neglected tropical disease (NTD) by the WHO, 42 of the 89 venoms were published, which nearly equals the number of all studies in the 15 years prior (Figure 1A) [71]. This reflects the increased awareness in science and public healthcare due to the increasing work of non-profit and governmental organizations, as well as the renewed interest in new antivenom approaches [72,73,74].

### 2.1. Meta Data of Investigated Snakes

The final venom composition of a study is strongly dependent on the examined specimens. Regarding this aspect, our analysis summarizes different information about the investigated snakes (Figure 1B–F).

The pool size is a parameter that helps to evaluate whether the study represents an average venom of a species, rather a few or only single individuals [22]. The usage of a pooled venom sample reduces the biological variance and increases the statistical power for the investigated population [123]. On the other hand, the analysis of non-pooled venoms from individuals allows for deeper insight into intraspecific variations, including the possibility to detect toxins of lower abundances, when signal intensity would otherwise be suppressed in a large pool size [22,123]. Interestingly, the exact number of pooled individual venoms was only provided for 48 proteomes, while the pool sizes for 41 proteomes were not specified (Figure 1B). The pool sizes range from a single examined specimen to up to 150 individuals, with a median of 8.5 investigated snakes in total, revealing a relatively small pool size. In the studies lacking pool size information, 10 out of 41 venoms reported at least a minimal pool size: >1, >5 or >10. The remaining 31 proteomes were without any information. Understandably, most of the latter venom samples were derived from commercial venom companies or were listed as derived from a serpentarium (Supplementary Material Table S1).

A second important aspect is the origin of the snakes. As mentioned above, the venom can differ in a single species between populations or collection areas. Providing geographical information is highly recommended to allow comparisons between different populations. From all 89 proteomes, we identified 42 which could be directly assigned to a specific region, whereas 32 only mentioned the country of origin and another 15 venoms provided neither a region nor a country (Figure 1C).

Finally, captivity is another factor discussed to influence venom composition. We summarized 40 proteomes that sourced on milked snakes in captivity, 31 from wild captures and 18 without information (Figure 1D). Commercially available venoms and gifts from institutes or antivenom manufactures were counted as ‘captivity’ sources if not stated otherwise. It is worth mentioning that sex as well as age, factors influencing the venom of snakes, are the least given information in proteomic venom studies. For 60 compositions, there was no information about the age and for 62 compositions no information about the sex could be found (Figure 1E,F). Regarding the age, all other proteomes were investigated from adult specimen (21) or different ages (7), and only one study directly compared and distinguished between the venom from juvenile and adult vipers [28].

### 2.2. Venom Proteome Data Accessibility

The deposition and storage of MS-based proteomics data in publicly accessible databases is of increasing importance and consequently several online platforms are nowadays available [124,125]. Remarkably, to date, the raw data of only 16 venom proteomes from 12 studies between 2016 and 2020 have been uploaded and are freely available. All uploaded proteomes were transmitted to the ProteomeXchange consortium for data repository either by PRIDE or massIVE [125,126,127]. The dataset identifiers, if mentioned in the respective study, are listed in Supplementary Material Table S1. Nevertheless, the comparatively poor number of uploaded proteome data reflects an enormous deficiency in general data accessibility within the snake venomics field.

### 2.3. Identified Toxin Families

Snake venoms are composed of a broad spectrum of enzymatic and non-enzymatic toxins affecting different biochemical targets. The origins of the components are a multitude of ancestral genes that were functionalized in the venom gland by duplication and neo- or subfunctionalization [20,128,129]. More than 20 different enzymatic and non-enzymatic protein and peptide families were described at the proteomic level from Viperinae venoms and are listed with their common abbreviations in Table 2. Among enzyme functions, this predominantly includes hydrolases (EC 3.-.-.-), but also oxidoreductases (EC 1.-.-.-) and transferases (EC 2.-.-.-).

The identified venom proteins can be classified regarding their average percentual occurrence into four groups: major, secondary, and minor toxin families as well as the kind of rare proteins that were observed only in a few proteomes. Toxins were clustered under their main family nomenclature because many studies did not subdivide respective observations within a single family into subfamilies, like svMP P-I, P-II and P-III or S49 and D49 PLA_2_. Further detailed information, complementing the identified subfamilies, are listed in Supplementary Material Table S1. Since a detailed examination of each individual toxin (sub)family would go beyond the scope of this review, a variety of reviews and papers are provided in the references section of Table 2.

The four major toxin families (svMP, PLA_2_, svSP, and CTL) dominate the picture of Old World viper venoms, which explains the hemorrhage and cytotoxic character of most viper envenomation [12]. Neurotoxic clinical profiles mainly caused by PLA_2_ are less common, but frequently reported for certain species [130,131]. In summary, the major toxin families account for 60–90% of venom compositions, with an average of 75%. The svMP family is the only consistently described toxin among all studies, while CTL is still present in 90% of the compositions. Three of the four major toxin families are enzymes, whereas the secondary toxin families (DI, CRISP, VEGF, and KUN) with the prominent exception of LAAO, mainly display non-enzymatic functions and represent 6–35% (average 17%) of venom compositions. Reported in a higher number of studies, the LAAOs showed a lower percentage compared to DI and are therefore listed behind DI. The minor toxin families (NGF, 5N, PDE, HYAL, PLB, and the *Bitis* genus unique CYS) were observed in less than half of the proteomes and with a total average share of 13%.

A limited number of toxin families was reported only for a single or few species and grouped as rare toxin families (average < 1%). These included the more abundant QC and AP, but also various other protease classes and even three-finger toxins (3FTx), which will be discussed in more detail below. Additionally, the occurrence of peptides is described in 45 venoms. These polypeptides mostly with masses <8 kDa often represent proteolytic products of C-type natriuretic precursor peptides including tripeptic svMP-i and the blood pressure decreasing BPP. In general, peptides as well as minor and rare toxin families are the least investigated parts of snake venoms, not only in Viperinae [132,133,134].

## 3. Venom Variations of Old World Vipers

In addition to the identification of specific proteins and their families in different viper species, the differences between toxin abundances are of great interest. Furthermore, knowledge on the venom composition is key to understanding not only the clinical profiles of snakebite envenomation, but also how environmental pressure possibly shaped venom compositions. To correlate these, it is important to consider the performed analysis type. Since the employed quantification approach varies from study to study, and also underlies other variabilities, this will almost certainly impact the general comparability.

### 3.1. The Bias of Quantification

A quantification approach in the venomics field gives insight into the abundance of toxins and toxin families, either in absolute or relative numbers. In accordance with the aforementioned biological factors, many experimental aspects, such as instrumental implementation, applied protocols, or databases affect the apparent protein composition [46,193,194]. For example, a venom study of the south Indian *E. c. carinatus* underlines the general importance of a taxonomically specific but not too limited database of protein sequences for annotation. The identified toxin families and toxin abundances in this *E. c. carinatus* composition were significantly dependent on to the selected database, regarding taxonomic family, genus or species [101].

**Figure 2:**
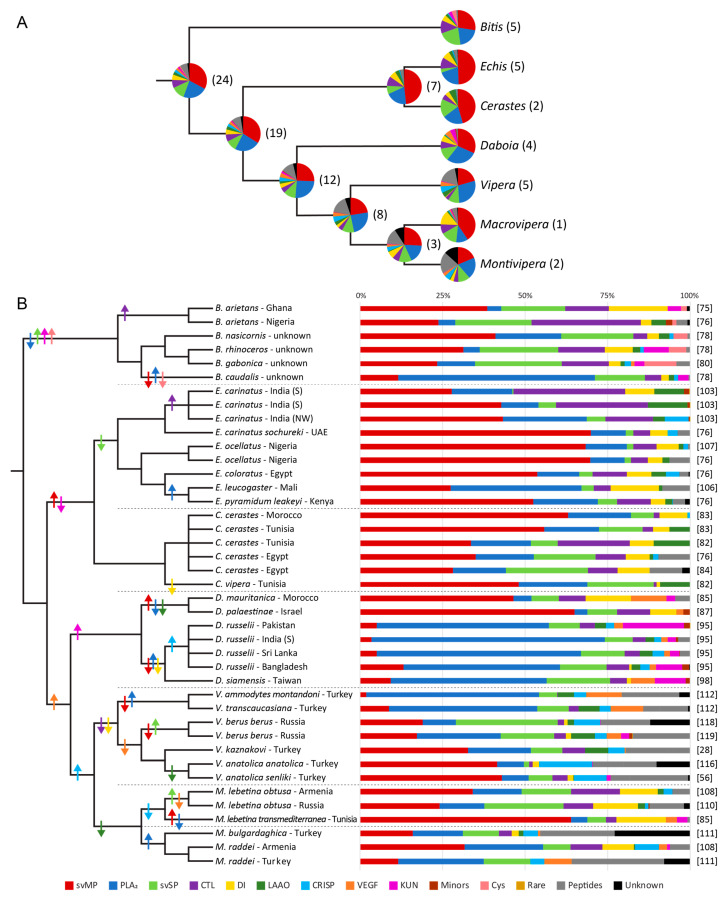
Snake venomics of Old World viper venom proteomes. (**A**) Overview of the genus venom compositions with the number of investigated species in brackets. Multiple studies per species have been equivalently summarized and used as a single species entry for the node compositions. Nodes were calculated in relation to the associated species numbers per branch. (**B**) Forty-one comparative proteomics data of 24 different Viperinae species and subspecies are lined up according to taxonomic relation and genera are separated by a dotted line. Origins of investigated specimen are mentioned after the (sub)species. Up and down arrows mark prominent abundance changes of a toxin family in the corresponding color to the previous node. The study references are listed behind the corresponding composition. Schematic cladograms of the phylogenetic relationships are based on phylogenetic studies mentioned in Materials and Methods (Section 5.2).

**Figure 3:**
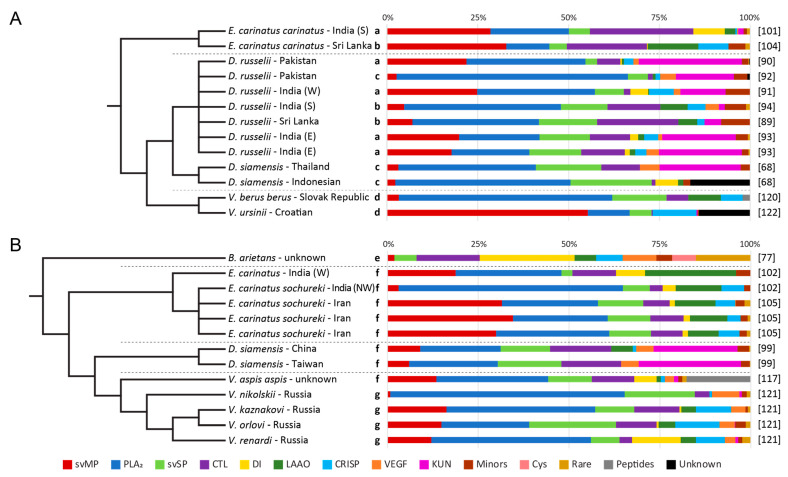
Old World viper venom proteomes by various quantification methods. (**A**) Five Viperinae species investigated by two-step quantifications and (**B**) 8 Viperinae species by whole venom in-solution shotgun approaches, with 13 proteomic data each. Used quantification methods are mentioned by bold letters: a (GF/EI+MSQ), b (1D SDS PAGE+MSQ), c (RP-HPLC+MSQ), d (2D SDS PAGE), e (emPAI), f (MSQ), g (MaxLFQ), with MSQ indicating MS-based quantification of spectral intensity or spectral count calculations. Species and subspecies are lined up according to taxonomic relation and genera are separated by a dotted line. Origin of investigated specimen are mentioned after the (sub)species name. The study references are listed behind the corresponding composition. Schematic cladograms of the phylogenetic relationships are based on phylogenetic studies mentioned in the Materials and Methods (Section 5.2).

Although all viper venoms were quantified by relative and label-free (without the usage of isotope or chemical labels) BU approaches and based on a tryptic digest, some workflow details strongly vary (Table 1). Relative protein abundances were either calculated from UV/Vis absorption at a certain wavelength, MS ion counts or by a combination of both. To minimize the effect of different detection and quantification methods, we clustered the quantifying studies into three groups (Figure 2 and Figure 3). A detailed overview of the applied workflows per venom composition can be found in Supplementary Material Table S1.

The most common method applied to true viper venoms is the so called ‘snake venomics’ approach, introduced by Calvete et al. in 2007, which is based on a three-level quantification protocol [46]. Accordingly, the relative abundance of a protein is calculated hierarchically regarding the reversed phase HPLC (RP-HPLC) peak area, 1D SDS-PAGE band intensity and if necessary, the TOP3 relative MS ion intensity.

In addition to the snake venomics approach, several other two-step quantification protocols are in use which are cumulated in a second group. This group consists of a preliminary physicochemical decomplexation step, followed by LC-MS/MS analytics. The separation by gel-filtration (GF), ion-exchange (IE), RP-HPLC or crude venom 1D-SDS PAGE is detected spectroscopically at different wavelengths. In the second step, after an enzymatic digest, the tryptic peptide abundance in a fraction/band is quantified by spectral intensities (SpI), spectral counting (SpC) or the average of both [195]. The combination of various separation methods and a MS-based quantification led to seven different protocols, not considering various normalization factors, like the number of observed or theoretically expected peptides or the molecular mass of the protein. In order to keep this simple, identical wavelength detection methods were summarized (Figure 3A(a–c)). Additionally, the two 2D-SDS PAGE densiometrically quantified and LC-MS/MS identified proteomes were also accounted to this group of two-step quantifications (Figure 3A(d)).

A third group of methods uses whole venom in-solution shotgun, short ‘shotgun’, approaches without additional decomplexation steps prior to LC-MS/MS analytics. These purely MS-based quantifications were calculated by similar SpI or SpC methods, but also the exponentially modified protein abundance index (emPAI) and accurate proteome-wide label-free quantification by delayed normalization and maximal peptide ratio extraction (MaxLFQ) were used [196,197]. The only study comparing two shotgun quantifications is from Kovalchuk et al. who showed that MaxLFQ and SpI-based quantifications render analogous percentual values [121].

All the above-mentioned differences underline the general challenge and complexity of such quantification protocols, which has here a particular focus on the snake subfamily of Viperinae. Considering other non-Viperinae venom studies would reveal even further workflows with major and minor differences. Another bias is the depth and comprehensiveness of the published analyses. While some studies mark non-annotated peaks or peptide-containing venom parts and include them in their calculation of the relative composition, others report only on the identified proteins. Others in turn, use a preliminary mass cut-off filter or a protocol-based detection limit, as e.g., one-step shotgun approaches only consider identified peptides. However, this type of calculation introduces a considerable bias into the true abundances of proteinaceous venom components. Accordingly, interstudy comparisons should always be handled with reservation if different quantification protocols are applied. The usage of more uniform protocols, or at least the online accessibility of raw data for individual reanalysis, as already mentioned, would be an important step to increase the comparability of quantitative data in the venomics field. Nevertheless, the comparison within the quantification groups allows for detailed insight into the venom compositions and trends of single species and genera.

### 3.2. Snake Venomics

The first group of snake venomics approaches includes 42 proteomes from 24 species. In two studies the identical venom pool sample was used and the more recent composition was included for direct comparison [82,85]. The remaining 41 proteomes are shown as a joint genus-wide comparison and the individual (sub)species level, related to their phylogenetic relationships (Figure 2). To avoid the statistical impact of highly investigated species due to several compositional datasets, a single species proteome was generated by their summed proteomes normalized to the total number of snake venomics studies for this species. These normalized proteomes were then handled equally to calculate the average genus compositions (Figure 2A).

The abundance of single toxin families can strongly differ between genera. However, as already mentioned, svMP and PLA_2_ in particular dominate the overall picture of the Old World viper venoms (Figure 2A). Other toxin families seem abundant in or even exclusive to some specific genera, like LAAO, CRISP, KUN, and CYS, which will be discussed in the following sections (Supplementary Material Figure S1). The mentioned bias of peptides and not-annotated venom parts are highlighted for the *Vipera* and *Montivipera* genera, with a high impact on the relative abundances of the other toxin families.

The dominance of smaller molecular masses and peptides, with often unknown physiological effects, described in only parts of the Old World vipers, shows that peptidomics of the non-classically defined toxins families is still an emerging field [198]. In particular, the combination with TD measurements or intact mass profiling (IMP) can compensate for existing gaps and give a detailed view on the venom content in the low molecular mass range [52,53].

### 3.3. Clade of African Adders

The venom of the African adders, *Bitis* genus, is mainly composed of different svMP (28%), like dimeric svMP-PIII, and compared to all other Viperinae they show the highest amount of svSP (22%) and CTL (12%) (Figure 2A). Members of the *Bitis* genus are the only Old World vipers containing CYS (4%) in their venoms (Supplementary Material Figures S1 and S2). While this protease inhibitor family is more prominent in the evolutionary distinct elapid venoms, CYS was also observed in the venom gland transcriptomes of species of the Viperinae subfamily *E. coloratus*, *V. kaznakovi*, and *V. anatolica senliki* [28,56,78,199]. The venom of *B. caudalis* is the only *Bitis* species that lacks on CYS and is dominated by a diversity of PLA_2_ (60%), forming high aggregate multimers compared to the monomeric ones in the other *Bitis* venoms (4–20%) [78]. This might be also due to the fact that the small *B. caudalis* belongs to the dwarf adder *Calechidna* subgenus, while the others are large-bodied adders of the subgenera *Bitis* and *Macrocerastes* [200]. Like *B. caudalis*, the venom of *B. arietans* is more divergent compared to all other *Bitis* species [78]. The major difference between *B. arietans* and the other *Bitis* venoms is the occurrence of the long-chain DI Bitistatin (18%) compared to the dimeric DI (2–9%) [75,78]. Studies also mark strong variations in the neutralization effect by polyspecific antivenoms against this genus [201,202,203].

### 3.4. Clade of Echis and Cerastes

With 15 proteomes from 7 species, the sister-groups *Echis* and *Cerastes* have been well studied by the snake venomics approach and allow for a good comparison of inter- as well as intraspecific aspects. Interestingly, the venoms of these vipers, on average, have the highest amounts of svMP and LAAO, while they are exclusively missing VEGF and KUN (Figure 2A).

Saw-scaled vipers, *Echis* genus, are known for their wide distribution from the western African coast, over the Arabian Peninsula to Sri Lanka and Bangladesh [5]. The intragenus and -species venom variations have been investigated in detail for *E. carinatus* [23,76,103]. Among all other vipers *Echis* on average displays the highest svMP content (50%), with strong percentual variations between the different species and regions of origin. *E. ocellatus* and *E. c. sochureki*, showed by far the highest svMP content (69–70%) of all Viperinae species. This is contrasted by only 27% svMP for the south Indian *E. carinatus* and Malian *E. leucogaster* venom (Figure 2B). This svMP dominance recently made them the target for a new antivenomic approach employing various metal chelators as complexants for cations of the active site from the zinc-dependent svMP [106]. Further, svSP (4%) showed the lowest abundance in the *Echis* genus and even further proteases were only observed as a very minor component, e.g., aspartic protease (<0.2%, rare protein families) (Supplementary Material Table S1). Overall, the venom profiles of the south Indian *E. carinatus* venoms with the large share of LAAO and CTL can be separated from the western distributed *Echis* species.

The North African desert vipers, *Cerastes* genus, have a simpler venom profile consisting of only seven toxin families (four majors, three secondaries) and several smaller peptides (Table 1). The venoms are based on svMP (46%) and PLA_2_, the latter with 18 ± 2% are nearly similarly abundant in all six *Cerastes* proteomes, albeit with remarkable differences in CTL and LAAO contents (Figure 2). With five different proteomic compositions dedicated to *C. cerastes*, this species is the best investigated by snake venomics approaches. Comparing the three regions of origin (Morocco, Tunisia, and Egypt) essentially covering North Africa, it seems that different venom populations of *C. cerastes* exist with a contact zone around Tunisia: an eastern lineage with a high amount of CTL (9–24%) and a western lineage with an increased amount of svMP (56–63%) and lower CTL (2–3%) [84]. The appearance of such kind of west and east lineages was already observed at a phylogenetic level of *C. cerastes* [204,205]. Remarkably, however, the immunorecognition capabilities of two antivenoms were similar for the *C. cerastes* venoms of all three regions and a low cross-reactivity against *C. vipera*, with a composition more similar to the western, than the eastern, *C. cerastes* [83]. The sole appearance of CRISP in the Tunisian venoms of *C. cerastes* as well as *C. vipera*, suggests a more complex distribution of venom differences in the *Cerates* genus. Due to a still comparatively low number of studies, this assumption needs to be further investigated.

### 3.5. Clade of Eurasian Vipers

Four genera (*Daboia*, *Vipera*, *Macrovipera*, and *Montivipera*) constitute the group of Eurasian vipers. With several subgenera, species, and subspecies, their taxonomic assignments have been thoroughly discussed [206]. Their venoms are highly diverse, and except for some rare toxins like PLA_2_ inhibitors or CYS, comprise representatives of each toxin family (Table 1 and Figure 2). The VEGF (1–6%) are restricted to Eurasian vipers, with only a low occurrence in *Bitis* venoms (0.2%) (Supplementary Material Figure S1). Most secondary toxin families are highly abundant in one genus or another compared to all Old World vipers: *Daboia* compositions have the highest occurrence of KUN (6%), *Vipera* of CRISP (6%), and *Macrovipera* venoms are richest in DI (13%). This finding of clear intergenus differences may be an indicator of evolutionary developments in the compositions (Figure 2A). Furthermore, large variations in the major toxin family ratios between the genera, especially among LAAO, DI, and CRISP are notable (Figure 2B). A problem for the direct comparison of compositions however is the percentage of neglected venom components. Previous discussed genera rarely include non-annotated peaks into their calculations of the venom composition and thus discriminate sizable amounts of peptides, mostly not visible on SDS-gels. In contrast, more recent studies on *Vipera* and *Montivipera* venoms, performed by Intact Mass Profiling overcome this bias and reflect a more accurate picture of the compositions.

The *Daboia* vipers can be phylogenetically divided into a western Mediterranean group and an eastern tropical Asian group, with two species each [206]. The comparison shows that also their venom compositions cluster well according to these lineages (Figure 2B). While the Mediterranean *D. mauritanica* and *D. palaestinae* are rich in svMP, increased in their DI and CTL content, and appear more closely related to *Macrovipera* venoms, the Asian *D. russelii* and *D. siamensis* venoms are high in PLA_2_. In particular, PLA_2_ are a protein family with various physiological profiles, such as cytotoxic and neurotoxic activities. This is also the reason for similar diverse pathological courses and lethal potential reported for *Daboia* envenomation, which are often grouped according to their geographic variations [95,98,207]. The physiological effects of PLA_2_ in the Asian *Daboia* venom reach from strongly anticoagulant but weakly lethal (e.g., RVV-VD) to neurotoxic and highly lethal (e.g., Drk-a1); hence neurotoxic effects are only clinically significant for *D. russelii* bites from south India and Sri Lanka [95,208]. Interestingly, the svMP RVV-X and the svSP RVV-V can be found in the venom of both Asian *Daboia* species, however, they have not been reported for the Mediterranean *Daboia* venoms. This might be because both venoms of *D. mauritanica* (former *Macrovipera mauritanica*) and *D. palaestinae* (former *Vipera xantina palaestinae*) were studied in comparison to other snake genera. A reanalysis with a more recent *Daboia* database would likely yield better insight in terms of comparability. Nevertheless, the venom of *D. mauritanica* is described with high similarities to the *M. lebetina transmediterranea* venom [85]. The east–west venom dichotomy within the *Daboia* genus is supported by the following points: The prominent DI (8–14%) in the Mediterranean *Daboia* spp. (Viperistatin) were only observed in traces (1%) for the Asian *Daboia* spp. (Russelstatin). Additionally, the KUN content with 2–18% is more abundant in *D. russelii* and *D. siamensis* compared to the other two (>3%) with several unique sequences between the analyzed regions [95].

The common vipers, *Vipera* genus, comprise 21 different species and a multitude of subspecies. The taxonomic diversity is also reflected by their venom compositions. The genus has the highest CRISP (6%) and lowest DI (>1%) occurrence among all Viperidae, and while some representatives have a high svMP content, others are rich in PLA_2_, svSP or CRISP (Figure 2B). Apart from this frame, some species show high PLA_2_-based venom compositions, e.g., *V. a. montandoni* and *V. transcaucasiana* (45–52%; e.g., Vaspin, Vipoxin, and Ammodytin variants), with a high level of VEGF (10–11%) which therefore resembles the *D. siamensis* venom. However, both toxin families are less abundant in the other five proteomes. Interestingly, the amount of svMP increases with a decreasing level of PLA_2_, and for both *V. b. berus* proteomes an increasing svSP content is observed. On the other hand, the two *V. anatolica* venoms are notable with their rich CRISP (10–16%) composition, low in LAAO and the lack of VEGF (Supplementary Material Figure S1).

From an analytical point of view the *Vipera* genus experienced the most TD investigations, which might be a reason for the high number of annotated peptides (Table 1). This includes a remarkable amount of svMP-i (6%), like the tripeptides pEKW and pENW, known for their occurrence in svMP-rich venoms. Interestingly, despite their low level of svMP, svMP-i were also identified in the proteomes of *V. a. montandoni* and *V. transcaucasiana*. For *V. a. montandoni* the svMP-i content is 11%, almost six times higher than the svMP content (2%) [112,187]. Furthermore, the *V. kaznakovi* study is the only one looking at the venomic specimen level of the analyzed pool, and thus highlights differences between individual snakes on the level of svSP and CTL as well as LAAO and PLA_2_, regarding age and sex [28]. This underlines the importance of adequate venom pool sizes of several specimens and is thus highly recommended for future venom proteomics studies in general.

The venom of the large Palearctic vipers, *Macrovipera* genus, has been quantified for only two *M. lebetina* subspecies: the northwest African *M. l. transmediterranea* and the Asian endemic *M. l. obstusa*. The surprisingly low venom similarity is mainly based on the DI contents, formed by either the dimeric Lebein or short Obustatin [108]. The svMP, as the most abundant toxin family, is for *M. l. transmediterranea* composed of PIII, while PI leads in *M. l. obtusa*. Compared to *M. l. transmediterranea*, both *M. l. obtusa* compositions show strong differences in the amount of PLA_2_, svSP, and CTL (Lebecetin) (Supplementary Material Figure S1). The large geographic distance between these subspecies’ populations seems to be a reason for these striking differences. The *D. mauritanica* (former *Macrovipera mauritanica*) distributed in northwest Africa shows a higher venom similarity to the co-localized *M. l. transmediterranea*, with the main content of svMP (64–68%) mostly contributed by svMP-PIII, and the occurrence of VEGF in both venoms which has not been described for *M. l. obtusa*.

The Middle East mountain vipers, *Montivipera* genus, consists of eight species in total. However, with only two studied species it is one of the least investigated genera. The genus is rich in PLA_2_, svMP P-III, and like the *Vipera* venoms has a high CRISP level (5%). Remarkably, a high peptide content of 20% was reported, which mainly constitutes the directed cleavage products of natriuretic precursor peptides like CNAP in the molecular mass range of 3–4 kDa. The listing of all molecular masses obtained from IMP, like in other Turkish *Vipera* venom studies, leads to these high peptide numbers. Moreover, the *Montivipera* compositions should be handled with reservation due to the dominance of non-annotated proteins (>13%) (Figure 2B).

### 3.6. Other Quantification Workflows

In this section, two other groups of quantifying venomics studies will be discussed: the manifold ‘two-step quantification’ and the ‘shotgun’ approaches (Figure 3). Both approaches, which differ in their workflows and quantification, were less frequently used for the analysis of Viperinae venoms. Even if a direct comparison is not recommended, general trends can be observed within the genera (Figure 2B and Figure 3).

For example, the relative shares of toxin families within a genus are mostly similar across the different quantification groups: KUN are dominant in *Daboia*, and were only detected in low abundance in *Bitis*, *Echis*, and *Vipera* (Figure 3). Moreover, VEGF are nearly non-existent in *Echis* and CYS are exclusive to *Bitis*. The biggest variations in the MS-based quantified compositions are the lower svMP occurrence in *Echis*, and the remarkably higher content of KUN in *Daboia* venom, compared to the venom analysis by the snake venomics approach (Figure 2 and Figure 3). However, it cannot be excluded that this might be also an effect caused by the different investigated populations or venom pools.

### 3.7. Two-Step Quantifications

The second venom analysis group is various and consists of an application mixture by physicochemical separations, followed by a subsequent spectroscopically detection at specific wavelengths, and are combined with different MS quantification methods, like previously discussed, except the 2D SDS PAGE. Hence, due to these different workflows, comparisons within this group had to be considered with some reservation (Figure 3A).

Both venoms of *E. c. carinatus* show the occurrence of similar toxin families [101,104]. Compared to *Echis*, the two *Vipera* venoms (*V. berus berus*, *V. ursinii*), quantified by 2D SDS densiometry, are lower in CTL, DI, and KUN, but enriched in CRISP and LAAO [120,122]. Interestingly, these *Vipera* venoms differ strongly in an intragenus correlation. In detail, the Slovak *V. b. berus* venom is dominated by PLA_2_, while the Russian populations analyzed by the snake venomics workflow have a significantly higher svMP and svSP occurrence. The *V. ursinii* follows this trend of svMP-based *Vipera* venoms, like the more closely related *V. kaznakovi* and *V. anatolica*.

Toxin families within the *Daboia* genus are dominated by PLA_2_ and svMP, whereas the CTL content varies in strong dependence on the region of origin [68,89,90,91,92,93]. Interestingly, it seems that the amount of svSP slightly increases from the western to the eastern *Daboia* distributions and that KUN are less abundant in the most southern Asian populations (*D. russelii*: south India, Sri Lanka; and *D. siamensis*: Indonesia) (Supplementary Material Figure S2A). Both tendencies on the content of svSP and KUN have also been observed in other quantification approaches of *Daboia* (Figure 2B and Figure 3B).

### 3.8. Whole Venom in-Solution Shotgun

The third group comprises purely MS based in-solution shotgun approaches. Like the others, this group shows clear differences between the single genera and underpins most of the previously mentioned trends (Figure 3B).

The composition of *B. arietans* obtained by shotgun proteomics is rich in DI (26%) but does not contain any PLA_2_. This finding, as well as the nearly complete absence of svMP and the high abundance of 3FTx (15%) is uncommon within venoms of this genus [77]. Even 3FTx have been extensively characterized from Elapidae, only few are described for Viperinae, like *D. russelii* and *V. nikolskii*, and the occurrence in such high amounts is considered as atypical and a rare toxin family in viper venoms [190,209,210,211,212,213].

The *Echis* venoms were mostly investigated for *E. c. sochureki* [102,105]. It shows that the three Iranian populations are highly similar, and that the west Indian *E. carinatus* venom resembles the Iranian rather than the northwest Indian composition of *E. c. sochureki*. The northwest Indian derived venom from Rajasthan differs with a high PLA_2_ content of 62% from all other *E. carinatus* studies. This implied significant differences in median lethal doses between *E. carinatus* populations as well as antivenom neutralizing potency with regard to marketed polyvalent antivenoms [102].

The composition of the East Asian *D. siamensis* venom follows a *Daboia* pattern in all three quantification groups [99] (Figure 2B and Figure 3). With a commonly low content of CRISP (<1%) in all *Daboia* studies, *D. russelii* is the only genus member whose venom displays a higher CRISP level (1–7%) (Supplementary Material Figure S2B).

The venom compositions of all five *Vipera* are characterized by a mixture of mostly acidic and basic PLA_2_ (24–65%), while their svMP levels (1–16%) are lower compared to the other studies quantified by group one snake venomics [117,121]. The PLA_2_-dominated Russian *V. nikolskii* venom is similar to the previously mentioned Slovak *V. b. berus*, with variations in the secondary toxin families and a notable low abundance of >1% svMP [120]. This underlines the close relationship often referred to the Nikolsky’s Viper as a subspecies *V. berus nikolskii* [206]. Within the Russian *Vipera* the genetically closest related *V. orlovi* and *V. kaznakovi* also show the highest similarity. They differ in the PLA_2_ to svSP ratio, with a larger svSP abundance for *V. orlovi* (Figure 3B). The Russian *V. kaznakovi* shows strong differences to the Turkish specimens collected closely to the Turkish-Georgian border. In addition to the different quantification methods applied, the recently described polyphyletic character of Georgian and Russian *V. kaznakovi* populations within the *Pelias* group might be another reason for this intraspecific variation [206].

### 3.9. Non-Quantified Venom Compositions

The last group comprises 21 Viperinae venoms, which was not quantified by any of the aforementioned methods but also includes studies that purely counted 2D-SDS PAGE spots or summarized numbers of identified sequences. Due to the protein intensities, non-quantified images of SDS gels or chromatograms give a rough estimate about the quantitative compositions. Several other compositions belong to rarely described snake venoms in the literature. These four rarely investigated genera (*Atheris*, *Causus*, *Eristicophis*, and *Pseudocerastes*) contribute eight different species in three studies. Further venomics studies would be interesting for these underinvestigated proteomes.

The venoms of night adders, *Causus* genus, are of great interest. These (semi-)fossorial snakes are outliers in regard to morphological and ecological aspects of the classical viperid scheme, and their venom compositions are uncommon [81,214]. They consist mainly of LAAO and svMP, with low amounts of svSP and CRISP. The venom of *C. lichtensteinii* includes only four toxin groups and the *C. rhombeatus* venom has additionally a few PLA_2_. Variations at the proteoform level showed remarkable differences in cross reactivity antivenom tests against both species [81]. Nevertheless, night adders have the simplest reported venom composition of all Old World vipers until now, regarding the identified toxin families, and are the only genus lacking single standing DI (Table 1). It is worth mentioning that the peptidome (MW < 10 kDa) is still unknown and might include additional and novel toxins.

The African bush vipers, *Atheris* genus, have a dominating PLA_2_ and DI content in their venom with a strong species-specific diversity at high molecular toxins range (30–70 kDa), like svMP and svSP [69]. Size-exclusion chromatograms reveal a higher correlation between *A. squamigera* and *A. nitschei* than to *A. chlorechis*. However, since only three of the 18 *Atheris* species have been examined at the proteomic level, most of the venom compositions are still unknown.

The three Middle Eastern vipers of *Eristicophis* and *Pseudocerastes* show extreme venom variations [70]. The venom of *E. macmahoni*, from the monophyletic leaf-nosed viper genus, is such an example for a broad diversity of its composition. On a 2D SDS gel it showed >160 proteoform spots, belonging to a mixture of all major and secondary toxin families, except for KUN, and without any minor nor rare toxin families. Kallikrein-like svSP and PLA_2_ spots are of dominant intensity and pattern. The two false horned vipers, *Pseudocerastes* genus, are different to *Eristicophis* in their venom compositions, regarding the lower number of 38–44 2D gel spots, with nearly no proteins in the range of 16–60 kDa [70]. The hemotoxic *P. persicus* venom is dominated by svMP P-III, PLA_2_, and CTL, while it is known for *P. fieldi* that its bite causes strong neurological effects [215,216]. Accordingly, neurotoxic PLA_2_ are most abundant followed by VGEF, NGF, and KUN, in addition to a few other toxins in traces.

The proteomic analysis of *B. arietans,* *B. gabonica*, and the Turkish *M. l. obtusa*, revealed similar toxins families, with regards to earlier discussions of these genera [60,79,109]. The *Vipera* genus was further investigated by four compositions of the two European Nose-horned viper subspecies *V. a. ammodytes* and *V. a. meridionalis*. They are rich in PLA_2_ (Ammodytins), followed by svSP, LAAO, CRISP, VEGF like Vammin and two recent studies described KUN in *V. a. ammodytes* venom as well [113,114,115]. They correspond to *V. a. montandoni* and *V. transcaucasiana*, which are discussed as subspecies *V. ammodytes transcaucasiana*, and underline the leading PLA_2_ trend in this clade [206].

The non-quantifying *Daboia* studies underline the division into an Asian and Mediterranean venom group, such as the svMP- and DI-rich Moroccan *D. mauritanica* venom [86]. In contrast, the venoms of the Indian *D. russelii* and *D. siamensis* (Myanmar and Taiwan) are dominated by PLA_2_, svSP, and KUN [88,97,100]. The earliest study from Nawarak et al. is difficult to classify since the databases in 2003 were much more limited in snake toxin sequences than they are today. Therefore, only PLA_2_ and a few proteases, including svMP, were described in addition to many unspecific hits [96]. Nevertheless, the two profiles of *D. siamensis* (formerly described as *D. russelii* subspecies) were similar and clearly set apart from other viper venoms of the Crotalinae subfamily and Elapidae.

## 4. Outlook

The in-depth analytical characterization of venom proteomes helps to assess the compositions in relation to geographic locations and trends between genera and lower taxon. This further aids in efforts for the development of more effective antivenom strategies [22,76,217]. Nowadays, after an envenomation, the administration of a mono- or polyclonal antivenom is the only effective clinical treatment. However, this classical antibody approach shows technical and safety issues, like impurities and batch-to-batch differences, particularly with regards to venom variations as a highly limiting factor [21,203,217,218]. Recent considerations in the next-generation antivenomics field are directed to overcome these obstacles by shifting the focus from species-specific, serum-derived to key toxin targeting, recombinant antibodies or the usage of non-antibody-based strategies [21,74,219].

We showed that the majority of Old World viper venoms are composed of a few toxin families (Table 1 and Table 2). Therefore, a general target-based approach would help to treat the major clinical profiles of envenomation. For example, small molecule inhibitors specific against svMP (Batimastat and Marimastat), PLA_2_ (Varespladib) or svSP (Nafamostat), the three most common toxin families in Viperinae, were already successfully tested against certain viperid venoms [15,220,221,222]. However, for many new potential antivenom therapies, clinical trials for their use against snakebites have not yet been carried out. Even so, several of these drugs have been advanced up to phase III in other indications or already have a validated safety profile in humans [220,223]. The development of such hybrid antivenoms composed of oligoclonal antibodies mixed with a universal toxin-family small molecule inhibitor cocktail, e.g., metal chelators, could be a new kind of broad-spectrum snakebite therapeutics [21,223]. The benefits of a global and not regionally limited ‘universal snake antivenom’ are manifold. The cost reduction due to big batch sizes and the international need would increase the availability in rural regions and tackle snakebites as a NTD.

A considerable number of viper genera were minimally investigated or not investigated at all, and it is estimated that still most of their venom compositions are unknown [102,224]. In the case of Viperinae, this particularly applies to the diverse *Atheris*, *Bitis*, and *Vipera*. Furthermore, in order to obtain more details about the venom compositions and variations, it is not only important to study more species, but also to investigate regional populations. For example, it would be interesting to analyze variations from a species like *V. berus*, whose populations can be several thousand kilometers apart. This gigantic distribution area also spans different climatic zones and offers a wide range of potential prey, factors which may have an impact on the venom composition.

In the coming years, it is expected that the number of Old World viper venom proteomes will exceed 100. Hence, the systematic compilation in databases and review articles, that summarize and list these data become of ever greater importance in the growing field of venomics. The investigation of further compositions and their variations in detail will help in the development of better snakebite treatments, provide new insights into venoms as an evolutionary model system, and eventually lead to the discovery of medically important treats in human healthcare.

## 5. Materials and Methods

To identify relevant publications for this review we investigated the genera, species, and subspecies of Old World vipers (Squamata: Serpentes: Viperidae: Viperinae) by an online search using the following search engines/databases and keywords, including a time limitation up to 31 December 2020.

### 5.1. Online Search and Selection Criteria

The PubMed database (https://pubmed.ncbi.nlm.nih.gov/) of the National Center of Biotechnology Information (NCBI) was used with “<*species*>/<*subspecies*>”. Google (https://www.google.com/) as well as Google Scholar (https://scholar.google.com/) were used with “snake venom composition (<*genus*>/ <*species*>/ <*subspecies*>)”, “snake venom proteomics (<*genus*>/<*species*>/<*subspecies*>)” and “snake venomics (<*species*>/<*subspecies*>)”. Additionally, the online databases of VenomZone (https://venomzone.expasy.org/) and the Snake Venomics Display were consulted [63]. The results were screened manually for proteomic studies. This includes the references of identified studies.

Publications showing only (RP-)HPLC profiles and/or crude venom 1D SDS-PAGE images, often in context of single fraction analysis against e.g., cancer cells or in antivenom studies, were excluded, if no proteomic analysis was performed. This also includes single toxin studies or partial venom characterizations.

### 5.2. Taxonomic Status and Phylogentic Relationships

The current genus, species, and subspecies status are based on The Reptile Database (http://reptile-database.reptarium.cz, accessed on 31 December 2020) and a list of all searched taxa can be found in Supplementary Material Table S2. For a proteome published under a since revised taxonomic name, it has been changed to reflect The Reptile Database status. The Supplementary Material Table S1 includes the (sub)species name of the original publication as well as the revised name for this review. The phylogenetic relationship between members of the Viperinae subfamily are mainly based on the study of Alencar et al. (2016) and for the Eurasian vipers (*Daboia*, *Vipera*, *Macrovipera*, *Montivipera*) on the recent work of Freitas et al. (2020) [1,206]. Further references were consulted for intragenus aspects [2,3,200,225,226].

## Figures and Tables

**Table 1:** Complete overview of the 89 Viperinae venom proteomes. The number of studied venom compositions per genus (grey), species, and subspecies (bracketed) are mentioned. Types of different sequence annotations (black bars) and quantification (black dot) are marked for each species. The checklist shows identified toxin families, sorted by their general abundance in the venom of all Viperinae into major, secondary and further toxin families (minor, rare families and peptides). Abbreviations: svMP (snake venom metalloproteinase), PLA_2_ (phospholipase A2), svSP (snake venom serine protease), CTL (C-type lectin-related protein), DI (disintegrins), LAAO (L-amino acid oxidase), CRISP (cysteine-rich secretory protein), VEGF (vascular endothelial growth factors F), KUN (Kunitz-type trypsin inhibitor), CYS (cystatin), svMP-i (svMP inhibitor), NP (natriuretic peptides).

										Majors				Secondaries											
Genus *Species*	No. of Compositions	Examined Subspecies	Bottom-Up	Top-Down	Edman Degradation	Q—Snake Venomics	Q—Two-Step Workflows	Q—in-Solution Shotgun	No Quantification	■svMP	■PLA_2_	■svSP	■CTL	■DI	■LAAO	■CRISP	■VEGF	■KUN	■Minor families	■CYS	■Rare Families	■svMP-i	■NP	■Other Peptides	References
*Atheris*	*3*																								
*A. chlorechis*	1		■						●	🗸	🗸	-	-	🗸	-	-	-	-	-	-	-	-	-	🗸	[69]
*A. nitschei*	1		■						●	🗸	🗸	🗸	-	🗸	-	-	-	-	-	-	-	-	-	🗸	[69]
*A. squamigera*	1		■						●	🗸	🗸	🗸	-	🗸	-	-	-	-	-	-	-	-	-	🗸	[69]
*Bitis*	*9*																								
*B. arietans*	4		■		■	●		●	●	🗸	🗸	🗸	🗸	🗸	🗸	🗸	🗸	🗸	🗸	🗸	🗸	🗸	🗸	🗸	[60,75,76,77]
*B. caudalis*	1		■		■	●				🗸	🗸	🗸	🗸	🗸	🗸	🗸	-	🗸	-	-	-	-	-	🗸	[78]
*B. gabonica*	2		■		■	●			●	🗸	🗸	🗸	🗸	🗸	🗸	🗸	🗸	🗸	-	🗸	-	-	🗸	🗸	[79,80]
*B. nasicornis*	1		■		■	●				🗸	🗸	🗸	🗸	🗸	🗸	🗸	-	-	-	🗸	-	-	-	🗸	[78]
*B. rhinoceros*	1		■		■	●				🗸	🗸	🗸	🗸	🗸	🗸	🗸	-	🗸	-	🗸	-	-	🗸	🗸	[78]
*Causus*	*2*																								
*C. lichtensteinii*	1		■						●	🗸	-	🗸	-	-	🗸	🗸	-	-	-	-	-	-	-	-	[81]
*C. rhombeatus*	1		■						●	🗸	🗸	🗸	-	-	🗸	🗸	-	-	-	-	-	-	-	-	[81]
*Cerastes*	*6*																								
*C. cerastes*	5		■	■	■	●				🗸	🗸	🗸	🗸	🗸	🗸	🗸	-	-	-	-	-	🗸	-	🗸	[76,82,83,84]
*C. vipera*	1		■		■	●				🗸	🗸	🗸	🗸	🗸	🗸	-	-	-	-	-	-	🗸	-	-	[82]
*Daboia*	*24*																								
*D. mauritanica*	2		■		■	●			●	🗸	🗸	🗸	🗸	🗸	-	-	🗸	🗸	-	-	-	-	🗸	-	[85,86]
*D. palaestinae*	1		■		■	●				🗸	🗸	🗸	🗸	🗸	-	-	🗸	-	🗸	-	-	-	-	-	[87]
*D. russelii*	12		■			●	●			🗸	🗸	🗸	🗸	🗸	🗸	🗸	🗸	🗸	🗸	-	🗸	🗸	🗸	🗸	[88,89,90,91,92,93,94,95]
*D. siamensis*	9		■		■	●	●	●	●	🗸	🗸	🗸	🗸	🗸	🗸	🗸	🗸	🗸	🗸	-	🗸	-	-	-	[68,96,97,98,99,100]
*Echis*	*16*																								
*E. carinatus*	11	(2)	■	■	■	●	●	●		🗸	🗸	🗸	🗸	🗸	🗸	🗸	🗸	🗸	🗸	-	🗸	🗸	🗸	🗸	[76,101,102,103,104,105]
*E. coloratus*	1		■		■	●				🗸	🗸	🗸	🗸	🗸	🗸	🗸	-	-	-	-	-	🗸	-	-	[76]
*E. leucogaster*	1		■			●				🗸	🗸	🗸	🗸	🗸	🗸	-	-	-	-	-	-	🗸	-	-	[106]
*E. ocellatus*	2		■		■	●			●	🗸	🗸	🗸	🗸	🗸	🗸	🗸	-	-	-	-	-	🗸	-	🗸	[76,107]
*E. pyramidum*	1		■		■	●				🗸	🗸	🗸	🗸	🗸	🗸	🗸	-	-	-	-	-	🗸	-	-	[76]
*Eristicophis*	*1*																								
*E. macmahoni*	1		■						●	🗸	🗸	🗸	🗸	🗸	🗸	🗸	🗸	-	-	-	-	-	🗸	-	[70]
*Macrovipera*	*5*																								
*M. lebetina*	5	(2)	■		■	●			●	🗸	🗸	🗸	🗸	🗸	🗸	🗸	🗸	🗸	🗸	-	-	🗸	🗸	-	[82,85,108,109,110]
*Montivipera*	*3*																								
*M. bulgardaghica*	1		■			●				🗸	🗸	🗸	🗸	🗸	🗸	🗸	🗸	-	-	-	-	🗸	-	🗸	[111]
*M. raddei*	2		■		■	●				🗸	🗸	🗸	🗸	🗸	🗸	🗸	🗸	-	🗸	-	-	🗸	🗸	🗸	[108,111]
*Pseudocerastes*	*2*																								
*P. fieldi*	1		■						●	🗸	🗸	🗸	🗸	-	🗸	🗸	🗸	🗸	🗸	-	-	-	-	-	[70]
*P. persicus*	1		■						●	🗸	🗸	🗸	🗸	🗸	🗸	🗸	🗸	🗸	🗸	-	-	-	-	-	[70]
*Vipera*	*18*																								
*V. ammodytes*	5	(3)	■	■	■	●			●	🗸	🗸	🗸	🗸	🗸	🗸	🗸	🗸	🗸	🗸	-	🗸	🗸	🗸	🗸	[112,113,114,115]
*V. anatolica*	2	(2)	■	■		●				🗸	🗸	🗸	🗸	🗸	🗸	🗸	-	🗸	🗸	-	🗸	🗸	🗸	🗸	[56,116]
*V. aspis*	1		■					●		🗸	🗸	🗸	🗸	🗸	🗸	🗸	🗸	🗸	🗸	-	🗸	🗸	-	🗸	[117]
*V. berus*	3		■		■	●	●			🗸	🗸	🗸	🗸	🗸	🗸	🗸	🗸	🗸	🗸	-	🗸	🗸	🗸	🗸	[118,119,120]
*V. kaznakovi*	2		■	■		●		●		🗸	🗸	🗸	🗸	🗸	🗸	🗸	🗸	-	🗸	-	🗸	🗸	🗸	🗸	[28,121]
*V. nikolskii*	1		■					●		🗸	🗸	🗸	🗸	-	🗸	🗸	🗸	🗸	🗸	-	🗸	-	🗸	-	[121]
*V. orlovi*	1		■					●		🗸	🗸	🗸	🗸	🗸	🗸	🗸	🗸	🗸	🗸	-	🗸	-	🗸	-	[121]
*V. renardi*	1		■					●		🗸	🗸	🗸	🗸	🗸	🗸	🗸	🗸	🗸	🗸	-	🗸	-	-	-	[121]
*V. transcaucasiana*	1		■	■		●				🗸	🗸	🗸	🗸	-	🗸	🗸	🗸	-	🗸	-	-	🗸	🗸	🗸	[112]
*V. ursinii*	1		■				●			🗸	🗸	🗸	🗸	-	-	🗸	-	🗸	🗸	-	🗸	-	-	-	[122]

**Table 2:** Toxin families in the venom proteomes of Viperinae. The proteins and peptides were grouped into families (bold) according to their general abundance in the venom compositions. LAAO and PLB were reported from more studies but show lower average percentages in the venoms than DI and HYAL. Enzymes are mentioned by their Enzyme Commission number (EC). Additional information about the average monomeric mass, number of disulfide bridges (No. of S-S) and their appearance in the proteomic studies is given. Asterisked entries mark that no exact number was derived from the literature. A more detailed list of the rare protein families is available in Supplementary Material Table S1.

Abbreviation	Snake Venom Toxin Family	Enzyme Class	Monomeric Size in kDa	No. of S-S	Observed in No. of Studies	References
**Major toxin families**						
svMP	snake venom metalloproteinase	EC 3.4.24.-	20–100	4–18	89	[135,136,137,138,139]
PLA_2_	phospholipase A_2_	EC 3.1.1.4	13–15	6–8	87	[140,141,142,143,144]
svSP	snake venom serine protease	EC 3.4.21.-	22–67	6	86	[145,146,147,148]
CTL incl. Snaclec	C-type lectin-related protein	-	13–15	3	80	[149,150,151]
**Secondary toxin families**						
DI	disintegrin	-	4–10	4–8	63	[152,153,154,155]
LAAO	l-amino acid oxidase	EC 1.4.3.2	50–70	2	68	[156,157,158]
CRISP	cysteine-rich secretory protein	-	20–33	8	63	[159,160,161]
VEGF	vascular endothelial growth factors F	-	10–15	5	48	[162,163,164]
KUN	Kunitz-type inhibitor	-	6–7	3	42	[20,165,166,167]
**Minor toxin families**						
NGF	nerve growth factor	-	12–37	3	41	[168,169,170]
5N	5′-nucleotidase	EC 3.1.3.5	73–100	4	34	[171,172]
PDE	phosphodiesterase	EC 3.1.4.1	90–140	16	33	[173,174]
HYAL	hyaluronidase	EC 3.2.1.35	33–110	5	17	[158,175,176]
PLB	phospholipase B	EC 3.1.1.5	~55	2	21	[177]
CYS	cystatin	-	12–15	2	8	[178,179,180]
**Rare families (selection)**						
QC	glutaminyl cyclotransferase	EC 2.3.2.5	33–40	1	17	[181,182]
AP	Aminopeptidase	EC 3.4.11.-	100–150	*	17	[183,184,185]
Peptides						
svMP-i	svMP-inhibitor	-	0.3	0	22	[134,186,187]
BPP	bradykinin potentiating peptide	-	0.5–1.5	0	24	[133,188,189,190]
other peptides	incl. further natriuretic peptides	-	1–10	-	24	[133,134,189,191,192]

## Data Availability

The data presented in this study are available in the Supplementary Materials section.

## Supplementary Materials

The following are available online at https://www.mdpi.com/article/10.3390/toxins13060427/s1, Table S1: Database of 89 Old World viper venom proteomes, Table S2: List of all searched taxa for the detailed literature search, Figure S1: Single toxin families by the snake venomics approach, Figure S2: Single toxin families by various quantification methods.
