# Supplementary material for: Old World Vipers—A Review about Snake Venom Proteomics of Viperinae and Their Variations
Source: Toxins (Basel). 2021 Jun 17;13(6):427. doi: 10.3390/toxins13060427 (PMC8235416; doi:10.3390/toxins13060427)
Supplement: Supplementary file 1 [file toxins-13-00427-s001.zip › toxins-1255510-SUPP/toxins-1255510-Supplementary.pdf]

Old World Vipers—A Review about Snake Venom Proteomics of Viperinae and their Variations

Maik Damm, Benjamin-Florian Hempel and Roderich D. Süssmuth

Abbreviations: svMP (snake venom metalloproteinase), PLA_2 (phospholipase A_2), svSP (snake venom serine protease), CTL (C-type lectin-related protein), DI (disintegrins), LAAO (L-amino acid oxidase), CRISP (cysteine-rich secretory protein), VEGF (vascular endothelial growth factors F), KUN (Kunitz-type trypsin inhibitor) and CYS (cystatin).

Table S1. Database of 89 Old World viper venom proteomes. All proteomes of Viperinae subfamily until 31 December 2020 are alphabetically listed by their current taxonomic status (see Materials and Methods). The original taxon of publications is mentioned. Additionally, details about the used venom pool, such as origin, age, and sex of the milked snakes, data accessibility of MS results by the studies, as well as the methods used for analysis and quantification for each proteome are listed. Detected toxin families and, if quantified, their percentual values or general observation in a venom are given, with a detailed annotation of detected minor and rare families. Cells with values in the analysis, quantification, and toxin family part are highlighted in green. The appearance of a toxin (sub)family without quantification is highlighted in orange.

Table S2. List of all searched taxa for the detailed literature search.

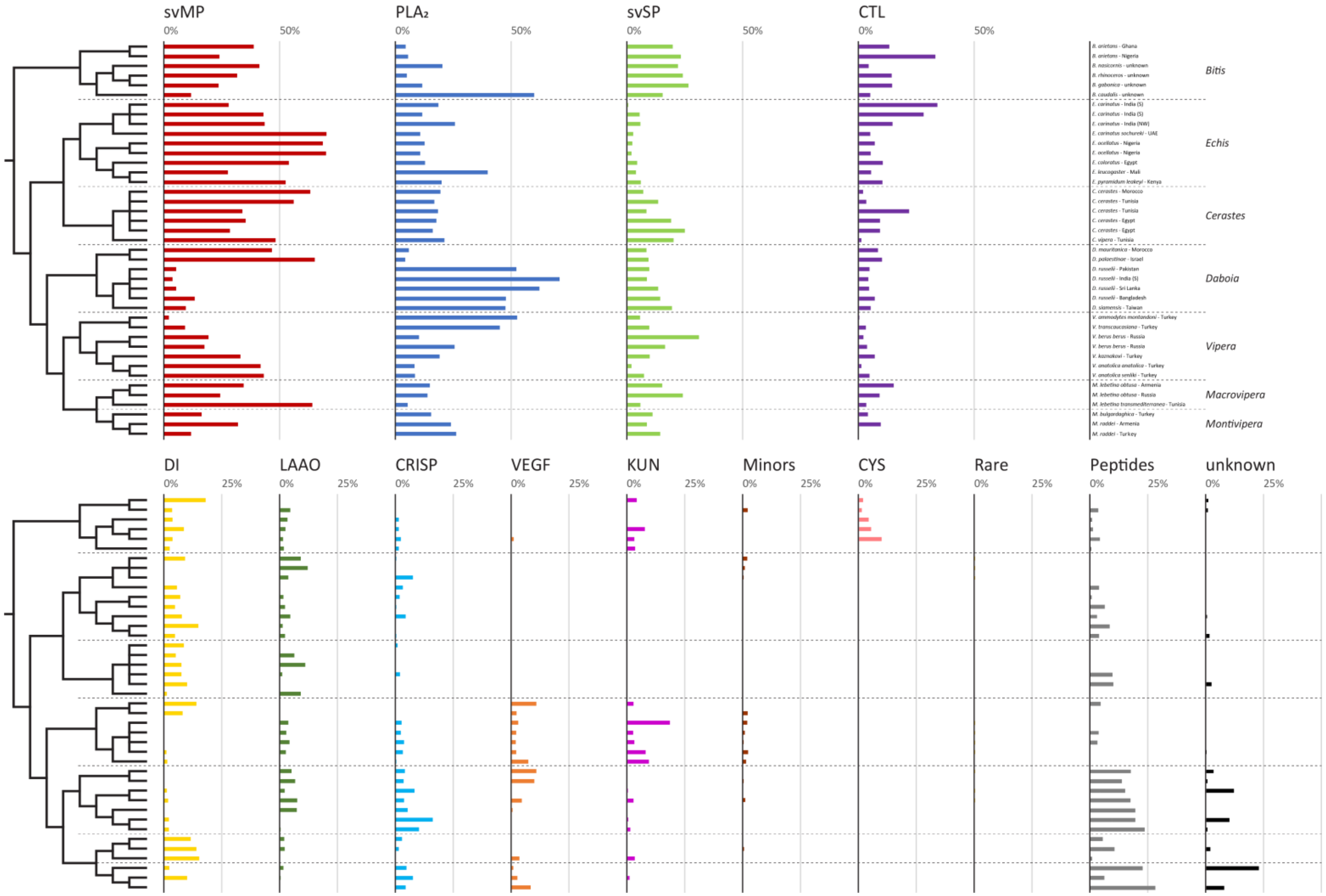

Figure S1. Single toxin families by the snake venomics approach. In total, 41 comparative proteomic data of 24 different Viperinae species and subspecies are lined up in a taxonomic relation and genera are separated by a dotted line. Origin of investigated specimen are mentioned after the (sub)species. Reference of study can be found in Figure 2B. Schematic cladograms of the phylogenetic relationships are based on phylogenetic studies mentioned in the Materials and Methods (Section 5.2.).

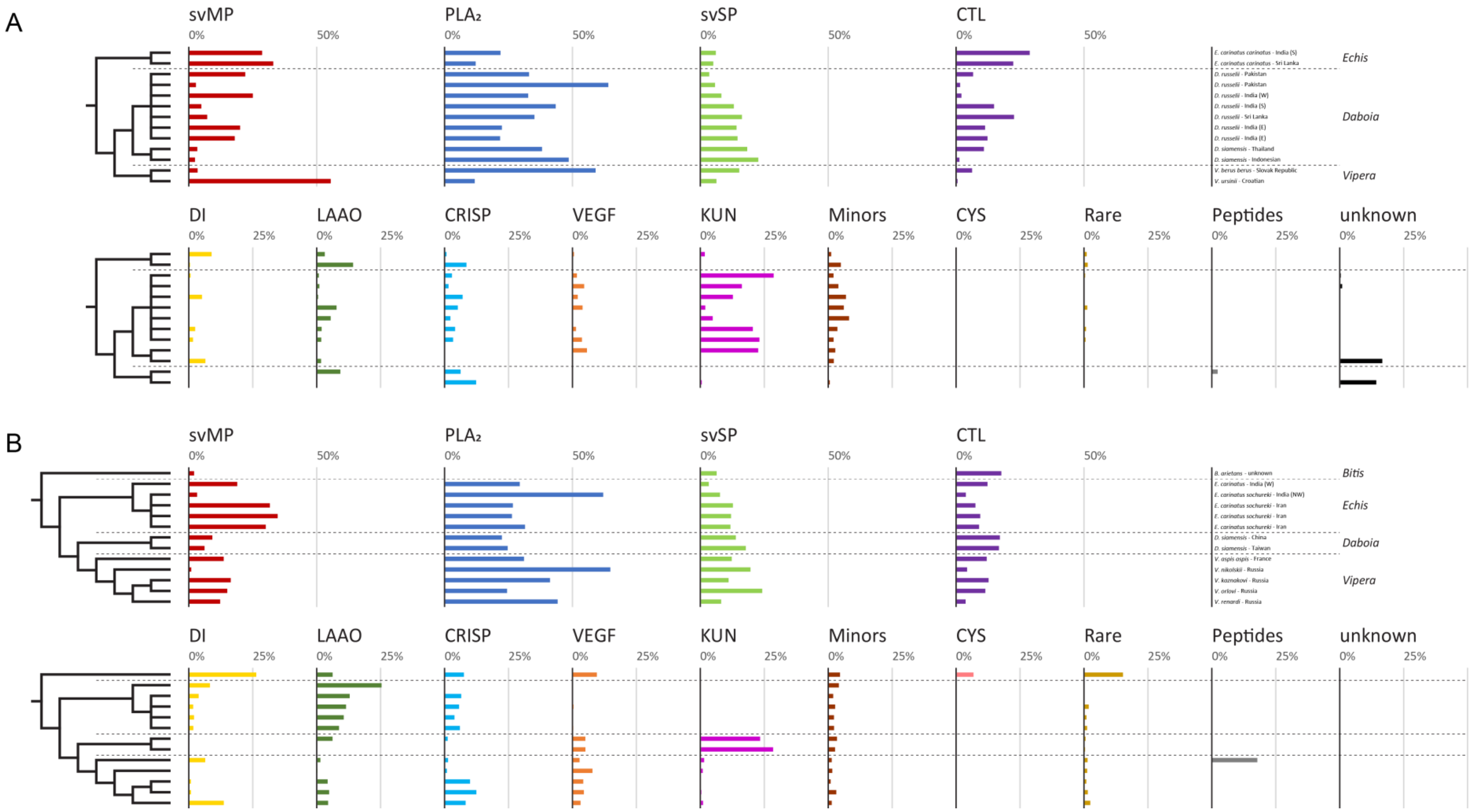

Figure S2. Single toxin families by various quantification methods. (**A**) 5 Viperinae species investigated by two-step quantifications and (**B**) 8 Viperinae species by whole venom in-solution shotgun approaches, with 13 proteomic data each. Species and subspecies are lined up in a taxonomic relation and genera are separated by a dotted line. Origin of investigated specimen are mentioned after the (sub)species. Reference of study can be found in Figure 3. Schematic cladograms of the phylogenetic relationships are based on phylogenetic studies mentioned in the Materials and Methods (Section 5.2.).
